# Impairing Eukaryotic Elongation Factor 2 Kinase Activity Decreases Atherosclerotic Plaque Formation

**DOI:** 10.1016/j.cjca.2014.09.019

**Published:** 2014-12

**Authors:** Peng Zhang, Maziar Riazy, Matthew Gold, Shu-Huei Tsai, Kelly McNagny, Christopher Proud, Vincent Duronio

**Affiliations:** a Department of Medicine, University of British Columbia and Vancouver Coastal Health Research Institute, Vancouver, British Columbia, Canada; b Biomedical Research Centre, University of British Columbia, Vancouver, British Columbia, Canada; c Centre for Biological Sciences, University of Southampton, Southampton, United Kingdom

## Abstract

We tested whether loss of eukaryotic elongation factor 2 kinase (eEF2K) activity in macrophages suppresses development of atherosclerosis by transplanting bone marrow from mice with mutant eEF2K into *ldlr*^*−/−*^ mice. Sixteen weeks after high-fat diet feeding, mutant eEF2K hematopoietic chimeras had a dramatically reduced level of atherosclerotic plaque formation. M1-skewed macrophages from eEF2K knock-in mice have less tumour necrosis factor-α release and a lesser ability to induce expression of endothelial cell markers, providing a potential explanation for the role of eEF2K. Because eEF2K activity in cells of the hematopoietic compartment contributes to atherosclerosis development, drugs inhibiting eEF2K might have a beneficial effect in treatment of atherosclerosis.

## Résumé

Nous avons vérifié si la perte d’activité kinase du facteur d’élongation-2 eucaryote (eEF2K) dans les macrophages inhibe le développement de l’athérosclérose en transplantant la moelle osseuse des souris eEF2K mutantes aux souris *ldlr*^*−/−*^. Seize (16) semaines après avoir commencé un régime riche en matières grasses, les chimères hématopoïétiques eEF2K mutantes ont présenté une réduction notable de la formation de la plaque d’athérosclérose. Les macrophages de type M1 des souris knoc-kin eEF2K présentent une moins grande libération du facteur de nécrose tumorale α et une moindre capacité à induire l’expression des marqueurs des cellules endothéliales, ce qui explique potentiellement le rôle de la eEF2K. Puisque l’activité de la eEF2K des compartiments cellulaires de l’hématopoïèse contribue au développement de l’athérosclérose, les médicaments inhibant la eEF2K auraient un effet bénéfique dans le traitement de l’athérosclérose.

Eukaryotic elongation factor 2 kinase (eEF2K) regulates protein synthesis via phosphorylation of its only known substrate, eukaryotic elongation factor 2 (eEF2)^1,2^; impaired binding of eEF2 to the ribosome and its dissociation from the ribosomal complex suppresses translocation of peptidyl-transfer RNAs.^3,4^ Numerous signalling pathways regulate phosphorylation of eEF2K at multiple activating or inhibitory sites, indicating its central role in regulating protein synthesis.^5-7^ eEF2K expression is upregulated in many cancers, and eEF2K promotes tumour survival by inhibiting protein synthesis during nutrient deprivation.^8^ eEF2K is also involved in neuronal development and synaptic plasticity.^9,10^ Deficiency of eEF2K activity induces fast-acting behavioural antidepressant effects, suggesting a potential use of eEF2K inhibitors as antidepressants.^11^ More recently, our laboratory and another have suggested that eEF2K is important in cardiovascular diseases.^12,13^

Atherosclerosis is recognized as a chronic inflammatory disease with a complex aetiology.^14,15^ Endothelial dysfunction, lipid retention, and an oxidative microenvironment are all thought to contribute to macrophage activation, survival, and/or proliferation and the subsequent development of early atherosclerotic lesions.^16^ A key mediator in the pathogenesis of this disease, oxidized low-density lipoprotein (oxLDL) has been shown to block apoptosis of macrophages through various signalling pathways.^17^

We showed that the eEF2K signalling pathway was activated by oxLDL.^12^ Impairment of eEF2K activity suppressed the prosurvival effect of oxLDL, suggesting eEF2K could influence the effect of macrophages on atherosclerosis. Using a targeted knock-in mouse strain with a mutation in the kinase-domain of eEF2K (*eef2k*-KI^18^), we investigated whether eEF2K deficiency affects atherosclerosis development in vivo in low-density lipoprotein receptor-deficient (*ldlr*^*−/−*^) mice. Wild type (WT) or *eef2k*-KI bone marrow was transplanted into irradiated *ldlr*^*−/−*^ mice. These hematopoietic chimeras were then fed a high-fat diet (HFD) for 16 weeks, allowing them to develop atherosclerosis. The extent of atherosclerosis was subsequently analyzed by en face staining of the aorta, and hematoxylin and eosin and Oil-Red-O staining of aortic root cross-sections. Our findings revealed a significant reduction in atherosclerosis in mice who received a transplant of bone marrow reconstituted from *eef2k*-KI mice, and thus revealed eEF2K as a potential target for therapy. We also report findings suggesting that tumour necrosis factor (TNF)-α release by M1-skewed macrophage cells is impaired when eEF2K activity is suppressed, thus providing a possible mechanistic explanation of the effects we have observed in vivo.

## Methods

### Mice

Generation of *eef2k*-KI transgenic knock-in (KI) mice have been described previously.^18^ These mice were crossed with WT C57BL/6 mice (strain code: 027, Charles River, Sherbrooke, Canada) for 6 generations to express kinase-inactive eEF2K in the C57BL/6 background. *ldlr*^*−/−*^ C57BL6 mice (strain name: B6.129S7-Ldlr^tm1Her^/J, stock number: 002207) were purchased from Jackson Laboratory. Mice were obtained and handled according to University of British Columbia ethics protocols A11-0141 and A10-0087.

### Bone marrow transplantation

The *ldlr*^*−/−*^ recipients were lethally irradiated using 14 minutes of exposure to 850-rad gamma irradiation (two 7-minute sessions). Donor bone marrow cells (2 × 10^6^) from either *eef2k*-KI or WT mice in 200 μL Hanks balanced salt solution were injected into the tail veins of the irradiated recipients. Chimeric animals recovered for 4 weeks before feeding the HFD. Animals were routinely monitored, and no major differences in weight or behaviour were noted between the groups.

### Atherosclerotic lesion analysis

Chimeric mice fed the HFD for 8 or 16 weeks were anaesthetized using intraperitoneal injection of 200 μL/10 g body weight of 2.5% avertin in phosphate-buffered saline (PBS), then perfused with 10 mL of 4% paraformaldehyde, 7.5% sucrose, and 0.5 mM ethylenediaminetetraacetic in PBS and flushed with 10 mL of 0.5 mM ethylenediaminetetraacetic in PBS. Hearts and aortas were harvested and stored in 10% formaldehyde. Lesion quantification was performed as described.^19^ For en face staining, aortas were opened longitudinally and pinned on a black wax pan for Sudan IV staining. For aortic root lesion quantification, the upper portions of the hearts were sent to Wax-It Histology Service Inc (Vancouver, British Columbia, Canada) for sectioning and hematoxylin and eosin and Oil-Red-O staining. The ratios of lipid-rich area to the total aortic area (en face staining) or the total lesion area (aortic sections) were calculated.

### Macrophage conditioned media preparation and assays

Bone marrow-derived macrophages were isolated from femurs of 6- to 8-week-old eEF2K-KI or WT mice as described^12^ and cultured in T75 tissue culture flasks for 11 days. Conditioned medium (CM) was harvested after 24-hour treatment with polarization factors: 10 ng/mL lipopolysaccharide plus 10 U/mL interferon-γ (for M1) or 10 ng/mL interleukin-4 (for M2), or from macrophages left in normal medium (M0 cells).

For endothelial cell response, early passages of human umbilical vein endothelial cells (HUVEC) were cultured in endothelial basal medium supplemented with EGM-2 BulletKit (Lonza, Mississauga, Canada). CM was applied to HUVEC cells at 80% confluency. After 5 hours, HUVEC cells were harvested for RNA extraction and subsequent quantitative polymerase chain reaction analysis for levels of vascular cell adhesion molecule-1 (VCAM-1) intercellular adhesion molecule-1 (ICAM-1), E-selectin, and P-selectin.

An enzyme-linked immunosorbent assay kit from BD Biosciences (Mississauga, Ontario, Canada) was used to test the CM for TNF-α. CM samples were diluted 100 times to ensure concentrations were within range of the standard curve.

## Results

**Figure 1:**
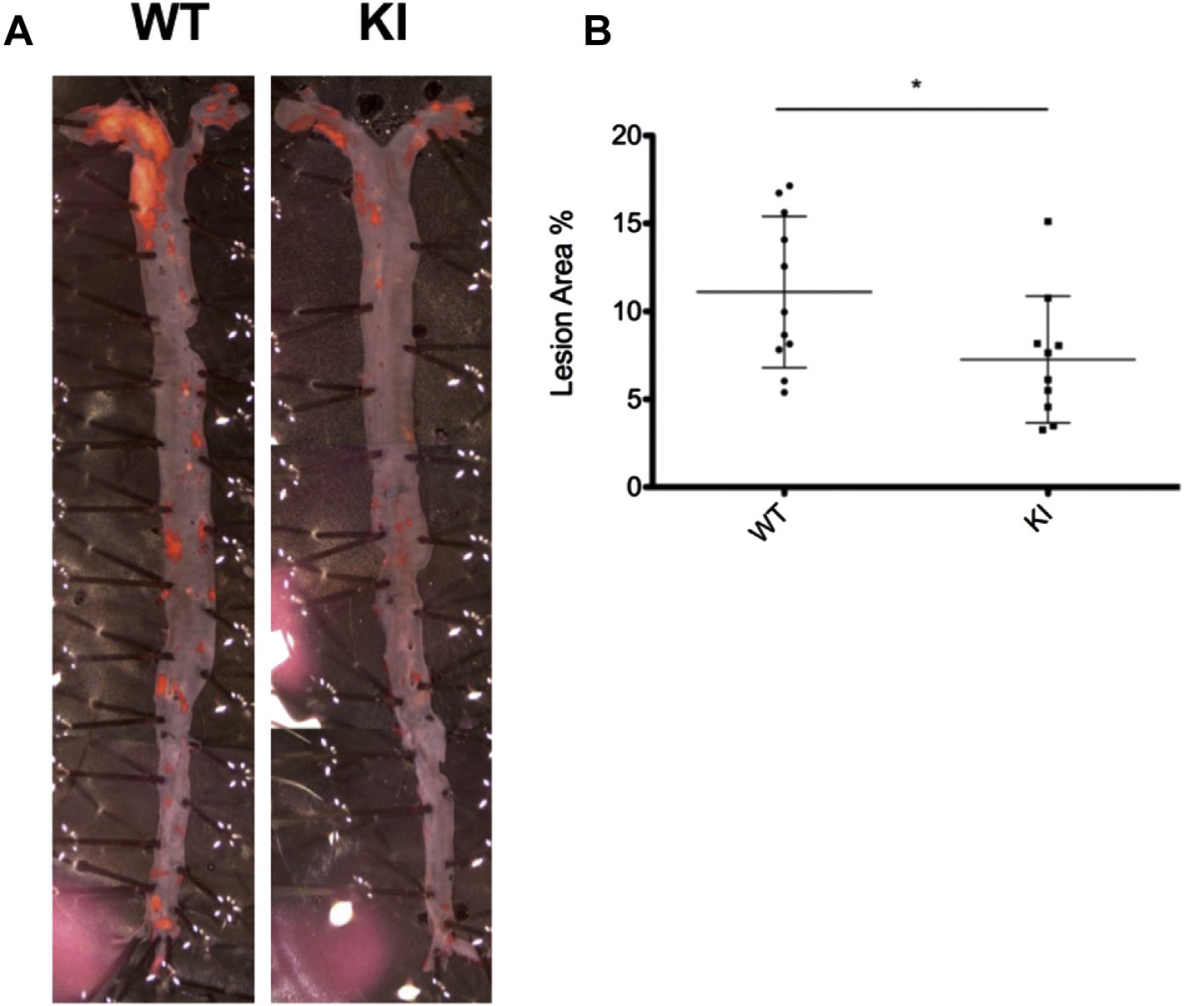
En face staining of plaque area after 16 weeks of high fat diet feeding. Chimeric *ldlr*^*−/−*^ mice were generated via lethal irradiation and transplantation with bone marrow from BL/6 (wild type [WT]) or *eef2k*-KI (knock-in [KI]) mice. Chimeric hearts were harvested after 16 weeks of high-fat diet feeding. (**A**) Representative images of Sudan IV-stained aorta show the difference in plaque area between WT and KI chimeric groups. (**B**) Quantification of relative plaque area (plaque area divided by total aorta area), which was performed by a blinded observer, shows that reduced activity of eukaryotic elongation factor 2 kinase in the hematopoietic compartment reduces plaque area from 13.0% (WT) to 7.7% (KI). WT: n = 12; KI: n = 10; *P* = 0.036, Student *t* test.

**Figure 2:**
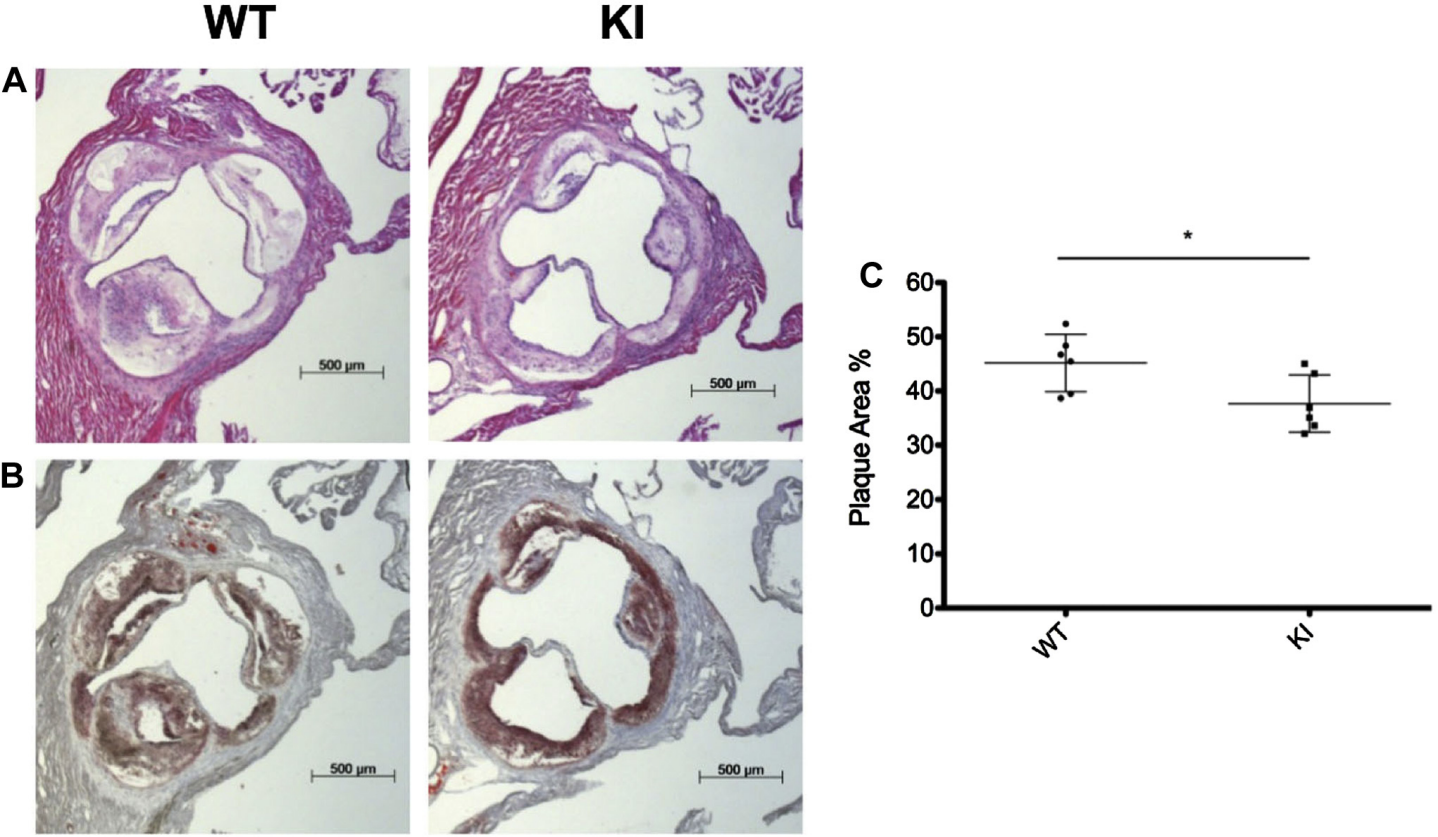
Plaque area in the aortic root after 16 weeks of high-fat diet. Chimeric animal hearts were harvested after 16 weeks of high-fat diet and compared with wild type (WT). Representative images of hematoxylin and eosin stained (**A**) and Oil-Red-O stained (**B**) aortic root show the difference of plaque area between WT and *eef2k*-KI (KI) groups. (**C**) Quantification of relative plaque area (plaque area over total aortic sectional area) in the aortic root, which was performed by a blinded observer, shows that reduced activity of eukaryotic elongation factor 2 kinase in the hematopoietic cells reduces plaque area from 45.2% (WT) to 37.7% (KI). WT: n = 6; KI: n = 6; *P* = 0.033, Student *t* test.

Hearts and aortas were harvested from chimeric mice, with bone marrow from either *eef2k*-KI mice or from WT BL/6 mice, after feeding a HFD for 8 or 16 weeks. En face staining of aortas in situ with Sudan IV was used to measure the lipid-containing surface area as a measure of the extent of atherosclerotic plaques. In mice who received a transplant of bone marrow from *eef2k*-KI mice, a striking reduction in lipid staining was observed after 16 weeks of receiving the HFD. Plaque areas were reduced from 13.0% (WT) to 7.7% (KI), a reduction of just > 40% (Fig. 1). Oil-Red-O staining was used to determine the extent of lipid deposits in aortic root cross-sections from harvested hearts. There was a significant decrease in plaque area in mice who received a transplant of bone marrow from *eef2k*-KI mice, 37.7%, compared with 45.2% in mice with WT bone marrow, also a statistically significant reduction (Fig. 2).

**Supplemental Figure 1:**
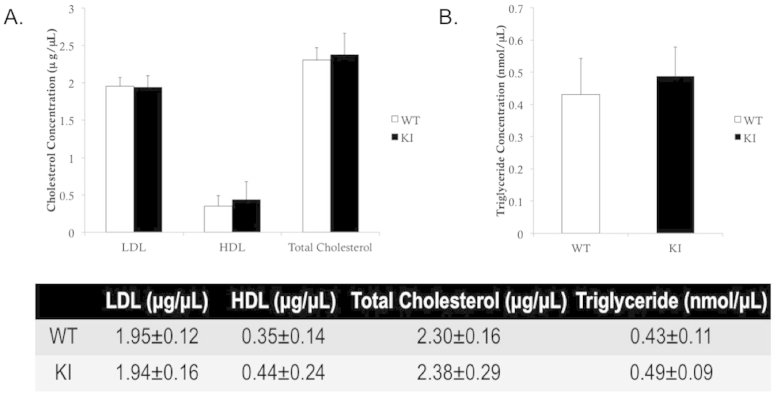


**Supplemental Figure 2:**
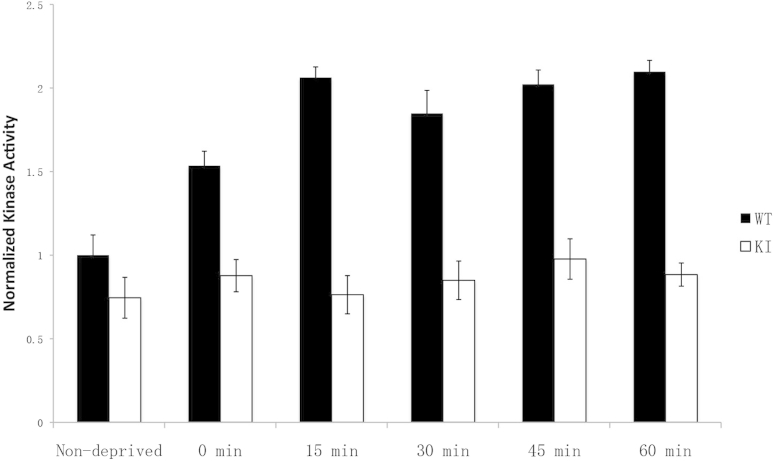


Although we saw a small decrease in atherosclerosis in the KI group after 8 weeks, it was not statistically significant (not shown), which might be attributed to the low degree of atherosclerosis observed at this very early time point. The possibility that the 2 groups of mice might have had different lipid profiles was ruled out (Supplemental Fig. 1). We also verified that macrophages isolated from the *eef2k-KI* mice had no detectable increase in eEF2 phosphorylation when stimulated with oxLDL (Supplemental Fig. 2).

**Figure 3:**
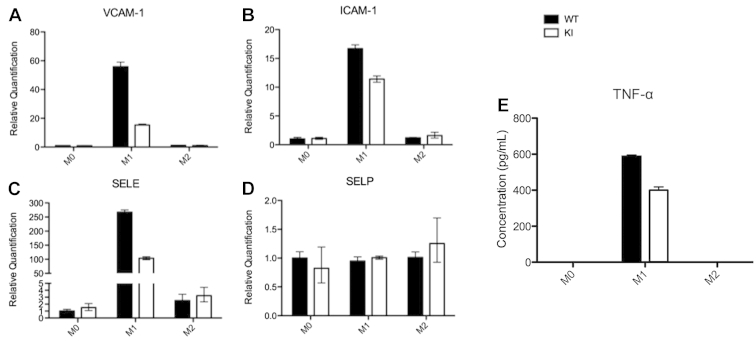
Lack of eukaryotic elongation factor 2 kinase activity compromises M1 cytokine-induced adhesion molecule expression in HUVECs. Macrophages were cultured in macrophage colony stimulating factor–containing media for 11 days. Cells were counted and seeded at the same concentration. Ten ng/mL lipopolysaccharide and 10 U/mL interferon-γ or 10 ng/mL interleukin-4 were added to polarize macrophages toward either the M1 or M2 subtype, respectively. Conditioned media (CM) was collected after 24 hours. VCAM-1 (**A**), ICAM-1 (**B**), E-selectin (SELE; **C**), and P-selectin (SELP; **D**) gene expression were determined in HUVEC cells incubated with the CM. CM from cells with inactivated eukaryotic elongation factor 2 kinase (KI groups) resulted in a reduced level of induction by the M1-conditioned media for VCAM-1, ICAM-1, and SELE, but not SELP. No significant changes were observed in the effects of CM from M0 or M2 cells. (**E**) After 24 hours, prepared CM was collected. Concentrations of tumour necrosis factor (TNF)-α were detected using enzyme-linked immunosorbent assay. Only M1 cultures produced significant levels of TNF-α, and M1 cells from *eef2k*-KI mice produced less TNF-α compared with the wild type (WT) cells. Results shown are from a single experiment using triplicate samples but the same trend was observed in 3 separate experiments for the HUVEC markers, but only a single experiment could be done for TNF-α analysis. HUVEC, human umbilical vein endothelial cells; ICAM-1, intercellular adhesion molecule-1; VCAM-1, vascular cell adhesion molecule-1.

As a source of cytokines, macrophages have paracrine effects on neighbouring cells, such as endothelial cells, and thus we tested if such activity might be altered by a deficiency in eEF2K activity. CM from macrophage cultures were tested for their effects on surface marker expression on endothelial cells. To mimic potential effects of inflammatory vs healer (M1 vs M2) macrophages, we compared macrophage CM from M0, M1, and M2 cells, each isolated from WT or *eef2k*-KI mice. CM from inflammatory M1 cells showed much greater activity than the others in inducing expression of E-selectin, VCAM-1 and ICAM-1. CM from similarly induced cells lacking eEF2K activity showed a marked reduction in activity (Fig. 3A-C). Levels of P-selectin expression were not affected by loss of eEF2K (Fig. 3D).

TNF-α is the key factor secreted by macrophages that regulate endothelial cell adhesion molecule expression. TNF-α concentrations in macrophage CM was highest in M1 conditioned media, as expected, and barely detected in M2 or M0 CM. Comparison of CM from WT or *eef2k*-KI cells showed that the TNF-α produced in the absence of eEF2K activity was reduced, matching the pattern of CM-induced adhesion molecule expression levels in endothelial cells (Fig. 3E).

## Discussion

Atherosclerotic plaques develop over an extended time period, with the initial processes at the endothelial lining causing activation of macrophages and their recruitment into the vessel walls, taking years (in humans) to cause any evidence of plaque formation. Macrophages are key players in that they respond to signals at the endothelial lining, and differentiate into foam cells as a result of accumulation of cholesterol and its byproducts from uptake of altered forms of low-density lipoprotein. We showed previously that survival of macrophages in the presence of oxLDL involves eEF2K, a regulator of protein translation.^12^ Based on the premise that macrophage survival contributes to their persistence in the intima, we postulated that blocking eEF2K activity might suppress development of atherosclerosis. We have tested this hypothesis in the *ldlr*^*−/−*^ mouse model by generating hematopoietic chimeras using bone marrow cells from mice expressing kinase-defective eEF2K (*eef2k*-KI) to engraft lethally irradiated *ldlr*^*−/−*^ recipients. After recovery, these chimeras were fed a high-fat diet to induce atherosclerosis and mice with mutant eEF2K had a greatly reduced level of atherosclerosis.

The ability of eEF2K to suppress protein synthesis, via phosphorylation of eEF2, is well characterized and is important in cellular responses to stress. Nutrient deprivation leading to activation of adenosine monophosphate-activated kinase, can contribute to eEF2K activation and the reduced protein synthesis serves as an important energy-saving mechanism.^8^ Before our study, no link had been reported between eEF2K activity and macrophage survival, and the current study emphasizes the potential importance of this pathway in the development of atherosclerosis. We have not yet been able to determine whether a difference in macrophage apoptosis can be detected in vivo, but this might be technically difficult, because a difference in apoptotic cells might only occur at early time points in disease progression, and it is well known that apoptotic cells are rapidly cleared in vivo. More studies will be required to investigate this question.

We have also shown that when eEF2K activity is impaired, the secretory profile of macrophages is changed, which might affect macrophage function in regulating the microenvironment and neighbouring cells (Fig. 3). We have assessed this function by measuring the expression of adhesion molecules, key factors that facilitate rolling and tethering of leukocytes, in HUVEC cells incubated with macrophage CM. Elevated levels VCAM-1, ICAM-1, and E-selectin were induced by TNF-α produced by M1 macrophages, and these proteins are known to result in enhanced progression of atherosclerosis.^20,21^ It should be noted that the lack of change in P-selectin message level might not be surprising because its regulation occurs mostly at the level of surface expression of preformed protein.^22^ M1 cells derived from *eef2k*-KI mice had considerably less activity in their CM, corresponding with a decrease in TNF-α.

Further characterization of macrophages lacking eEF2K activity, and analysis of changes in numbers or characteristics of macrophages that are present in plaques are the next steps in this project. We will also want to determine if suppression of endothelial cell surface proteins can be observed in the absence of eEF2K in vivo. Finally, it will be important to begin testing eEF2K inhibitors in vivo for their effectiveness in blocking atherosclerosis development. Although our studies show a partial block of atherosclerosis when eEF2K activity is suppressed, eEF2K inhibitors might become even more valuable when used together with other atherosclerosis drug therapies. Recent studies have implicated eEF2K activity in other conditions that also highlight its potential as an important drug target, including in cancer.^8^ In the future, it will be important to test newer specific eEF2K inhibitors in multiple animal models, to evaluate their potential as therapies for multiple disease targets.

## References

[1] Ryazanov A.G., Shestakova E.A., Natapov P.G. (1998). Phosphorylation of elongation factor 2 by EF-2 kinase affects rate of translation. Nature.

[2] Nairn A.C., Bhagat B., Palfrey H.C. (1985). Identification of calmodulin-dependent protein kinase III and its major Mr 100,000 substrate in mammalian tissues. Proc Natl Acad Sci U S A.

[3] Carlberg U., Nilsson A., Nygard O. (1990). Functional properties of phosphorylated elongation factor 2. Eur J Biochem.

[4] Browne G.J., Proud C.G. (2002). Regulation of peptide-chain elongation in mammalian cells. Eur J Biochem.

[5] Knebel A., Morrice N., Cohen P. (2001). A novel method to identify protein kinase substrates: eEF2 kinase is phosphorylated and inhibited by SAPK4/p38delta. EMBO J.

[6] Redpath N.T., Foulstone E.J., Proud C.G. (1996). Regulation of translation elongation factor-2 by insulin via a rapamycin-sensitive signalling pathway. EMBO J.

[7] Browne G.J., Finn S.G., Proud C.G. (2004). Stimulation of the AMP-activated protein kinase leads to activation of eukaryotic elongation factor 2 kinase and to its phosphorylation at a novel site, serine 398. J Biol Chem.

[8] Leprivier G., Remke M., Rotblat B. (2013). The eEF2 kinase confers resistance to nutrient deprivation by blocking translation elongation. Cell.

[9] Iketani M., Iizuka A., Sengoku K. (2013). Regulation of neurite outgrowth mediated by localized phosphorylation of protein translational factor eEF2 in growth cones. Dev Neurobiol.

[10] Chotiner J.K., Khorasani H., Nairn A.C., O'Dell T.J., Watson J.B. (2003). Adenylyl cyclase-dependent form of chemical long-term potentiation triggers translational regulation at the elongation step. Neuroscience.

[11] Autry A.E., Adachi M., Nosyreva E. (2011). NMDA receptor blockade at rest triggers rapid behavioural antidepressant responses. Nature.

[12] Chen J.H., Riazy M., Smith E.M. (2009). Oxidized LDL-mediated macrophage survival involves elongation factor-2 kinase. Arterioscler Thromb Vasc Biol.

[13] Usui T., Okada M., Hara Y., Yamawaki H. (2013). Eukaryotic elongation factor 2 kinase regulates the development of hypertension through oxidative stress-dependent vascular inflammation. Am J Physiol Heart Circ Physiol.

[14] Wong B.W., Meredith A., Lin D., McManus B.M. (2012). The biological role of inflammation in atherosclerosis. Can J Cardiol.

[15] Verma S., Gupta M., Ridker P.M. (2012). Therapeutic targeting of inflammation in atherosclerosis: we are getting closer. Can J Cardiol.

[16] Moore K.J., Tabas I. (2011). Macrophages in the pathogenesis of atherosclerosis. Cell.

[17] Hundal R.S., Salh B.S., Schrader J.W. (2001). Oxidized low density lipoprotein inhibits macrophage apoptosis through activation of the PI 3-kinase/PKB pathway. J Lipid Res.

[18] Gildish I., Manor D., David O. (2012). Impaired associative taste learning and abnormal brain activation in kinase-defective eEF2K mice. Learn Mem.

[19] Tangirala R.K., Rubin E.M., Palinski W. (1995). Quantitation of atherosclerosis in murine models: correlation between lesions in the aortic origin and in the entire aorta, and differences in the extent of lesions between sexes in LDL receptor-deficient and apolipoprotein E-deficient mice. J Lipid Res.

[20] Nakashima Y., Raines E.W., Plump A.S., Breslow J.L., Ross R. (1998). Upregulation of VCAM-1 and ICAM-1 at atherosclerosis-prone sites on the endothelium in the ApoE-deficient mouse. Arterioscler Thromb Vasc Biol.

[21] Dong Z.M., Chapman S.M., Brown A.A. (1998). The combined role of P- and E-selectins in atherosclerosis. J Clin Invest.

[22] Pan J., Xia L., McEver R.P. (1998). Comparison of promoters for the murine and human P-selectin genes suggests species-specific and conserved mechanisms for transcriptional regulation in endothelial cells. J Biol Chem.

