# Supplementary material for: Impairing Eukaryotic Elongation Factor 2 Kinase Activity Decreases Atherosclerotic Plaque Formation
Source: Can J Cardiol. 2014 Dec;30(12):1684–8. doi: 10.1016/j.cjca.2014.09.019 (PMC4424975; doi:10.1016/j.cjca.2014.09.019)
Supplement: Supplementary Data [file mmc1.pdf]

Supplementary Method

Lipid profile measurement

Blood for lipid measurements was drawn from the hearts before their recovery from mice. Concentrations of HDL, LDL, and Total Cholesterol were measured using HDL and LDL/VLDL Cholesterol Assay Kit (Abcam Cat. No. ab65390). Concentrations of triglyceride were measured using a Triglyceride Quantification Kit (Abcam Cat. No. ab65336).

Supplementary Results

To eliminate the possibility that the two groups of mice may have had different lipid profiles following their HFD feeding, we measured lipid components in the serum collected from the same mice used for *en face* staining and Oil-Red-O staining. All measures, including cholesterol (LDL and HDL) and triglyceride levels were not significantly different in the two groups of mice (Supplementary Figure 1), confirming that no metabolic or lipid uptake changes that could have confounded interpretation of the data.

To confirm that eEF2K activity was absent in macrophages from *eef2k-KD* mice, BMDM were isolated, as done routinely in our laboratory, deprived of M-CSF and then stimulated with oxLDL. Assay for phosphorylation of eEF2 showed increases in response to oxLDL treatment only in cells from wild type mice (Supplementary Figure 2).

Supplementary Figure 1.

Serum lipid profile following 16-week HFD. Blood samples were harvested from *ldlr*$^{-/-}$ chimeric mice after 16-weeks on a high fat diet. Low density lipoprotein (LDL), high density lipoprotein (HDL), total cholesterol (A) and triglyceride (B) in the serum were measured using colorimetric assays. No significant difference was observed between two groups (WT: n=12; KI: n=12)

Supplementary Figure 2

EEF2K Activity Assay. Macrophages are extracted from WT or KI mice and cultured for 7 days before treatment. MCSF was deprived in all groups except “non-deprived” for 4 hours. 25 ug/mL oxLDL was added to all groups except “non-deprived”. Cells were collected after time indicated. Phosphorylation of eEF2 was examined by western blot probed by p-eEF2 antibody. * $P<0.05$ ANOVA test.
